# Motor control strategies and the effects of fatigue on golf putting performance

**DOI:** 10.3389/fpsyg.2013.01005

**Published:** 2014-01-13

**Authors:** John F. Mathers, Madeleine A. Grealy

**Affiliations:** ^1^School of Sport, University of StirlingStirling, UK; ^2^School of Psychological Sciences and Health, Humanities & Social Sciences, University of StrathclydeGlasgow, UK

**Keywords:** motor control, scaling strategy, model, fatigue, golf putting

## Abstract

This study investigated the strategies used by elite golfers to scale their putting actions to achieve putts of different distances. There were three aims; to determine if putting actions are scaled by manipulating swing amplitude as predicted by Craig etal. (2000), to establish the test–retest reliability of the Craig et al. model, and to evaluate whether elite golfers changed their putting scaling strategies when fatigued. Putting actions were recorded at baseline (time 1) and 6 months later (time 2) and after walking at 70% of maximum heart rate for 1 h (time 3). Participants performed a total of 80 putts which varied in distance (1 m, 2 m, 3 m, and 4 m) at time 1 and time 2, and 100 putts to the same distances when they were fatigued (time 3). Multiple regression was used to examine how the golfers systematically changed the movement control variables in the Craig etal. (2000) model to achieve golf putts of different distances. Although swing amplitude was a strong predictor of putterhead velocity at ball impact for all of the participants at baseline (time 1), each golfer systematically changed aspects of the timing of their action. A comparison of the regression models between time 1 and time 2 showed no significant changes in the scaling strategies used, indicating that the Craig etal. (2000) model had good test–retest reliability. Fatigue was associated with a decrease in the number of putts that were successfully holed and significant changes in the scaling strategies used by three of the golfers, along with a trend for increasing the putterhead velocity at ball impact. These motor control changes in performance when fatigued were evident in successful putts indicating that even when these elite golfers were able to achieve the goal of holing the putt, moderate levels of fatigue were influencing the consistency of their performance. Theoretical implications for the Craig etal. (2000) model and practical implications for elite golfers are discussed.

## INTRODUCTION

Success in golf putting requires the golfer to perceive a putt distance and green characteristics (i.e., the speed of the putting surface, the degree of slope, and “borrow”) then translate this perception into an action where the ball is struck by the putterhead with an appropriate force and direction (Pelz, 2000; Penner, 2002; Hume et al., 2005). The ability to correctly judge the momentum to be transferred from the putterhead to the ball is critical in the elite game, but relatively few researchers have investigated this aspect of motor control in elite players and how the demands of competitive play impact on this skilled performance.

The way in which golfers adjust the putting stroke to achieve putts of different distances was investigated by Craig et al. (2000). Based on a kinematic analysis of the putting action they devised a model that could be used to describe how different putt distances are produced. If developed this model could have significant application for coaching practitioners and the research community as it may provide a description of how an elite golfer produces putts of different distances (their scaling strategy), the relative stability of this behavior, and whether certain scaling strategies make the golfer more susceptible to errors when they are in competitive situations. This could form the basis of an intervention procedure where a golfer’s scaling strategy is identified and then tested under different conditions to determine the extent to which it remains robust and effective under the physical and mental conditions of competitive play.

Craig et al. (2000) used a series of mathematical equations to demonstrate that the distance traveled by the ball is proportional to the squared velocity of the putterhead at impact ($V_{c}^{2}$):

(1) $$V_{c}^{2}=\left(2\lambda M_{c}/F\right)D^{2}\left(1/T^{2}\right){\left(P_{t}/k\right)}^{2}{\left(1-P_{t}^{2}\right)}^{\left(2/k\right)-2}$$

where *M*_c_ is the effective mass of the club-body system, *D* is the amplitude of the downswing, *P*_t_ is the proportion of the swing duration before the ball is struck, *T* the duration of the swing, *k* the point at which peak velocity occurs in the swing, and λ is a constant. Essentially, this equation proposes that a golfer alters the distance of their putt by systematically changing, or holding constant, the swing amplitude (*D*), the proportion of the swing duration before the ball is struck (*P*_t_), the duration of the swing (1/*T*^2^), or the time at which peak putterhead velocity occurs in the swing (*k*).

The method of scaling advocated by golf instruction textbooks suggests that the number of variables being systematically changed by any performer should be kept to a minimum, the logic being that consistent performance is more easily brought about by a simple model (Owens and Bunker, 1989; Pelz, 2000; Fairweather et al., 2002). Craig et al.’s (2000) original data set provided some tentative support for this simplicity principle and suggested that the most likely method of achieving various putt distances was by changing the amplitude of the downswing whilst keeping the other variables constant. They attempted to determine the method of scaling used by a group of golfers by plotting each of the four variables in the model against putt distance. On the basis of these plots they concluded that changing the distance of the putt was most probably accomplished by modulating the swing amplitude (*D*) since the other three variables (*P*_t_, 1/*T*^2^, and *k*) showed less systematic change, although no formal test of this was provided. Moreover, the Craig et al. (2000) study was carried out using non-elite golfers on an artificial putting surface where participants had to make the ball come to rest at a pre-determined position rather than striking each putt into a golf hole. This experimental design reduces the ecological validity of the performance task and the findings from the Craig et al. (2000) study may not reflect the elite performer’s habitual method of putting which may involve different motor control strategies (Masters, 1992; Pelz, 1994; Landsberger, 1998).

Whilst manipulating the amplitude of the swing provides a simple method of varying putt distance, other systematic variations in either *P*_t_,1/*T*^2^,or *k* could produce similar outcomes. These four variables could be manipulated individually or in combination with each other, to achieve different putt distances. For example, Fairweather et al. (2002) found that golfers scaled the putterhead velocity at ball impact for longer putt distances by increasing the amplitude of the swing and decreasing the duration of the swing. This interactive manipulation would translate into relatively small alterations to the movement variables and could preserve the proportion of the swing duration before the ball is hit (*P*_t_) and the shape of the velocity profile of the movement (*k*), resulting in them remaining relatively constant. However, it should be recognized that Fairweather et al.’s (2002) research was carried out on novice golfers and more elite golfers may be reluctant to modify the timing of their movement given the importance of the sequential organization of each segment, and their temporal relationships to each other. This would be consistent with previous work that has emphasized the importance of preserving the temporal relationships of intra-movement segments within movement scaling (Whiting, 1985), and by those who propose that overall movement duration is an invariant characteristic within a skilled movement class (Schmidt, 1975, 2003). To date, no research studies have clarified whether elite golfers alter their putting actions using a uniform method, or whether certain methods of scaling make the player more or less susceptible to making errors when they experience feelings of anxiety or fatigue. This is somewhat surprising given the on-going quest for valid, reliable, and sport specific performance assessments against which coaching and practice interventions can be evaluated (Bishop, 2008; Drust, 2010). Moreover, if competitive conditions are associated with changes in motor control in the elite player, it would be important to identify which of the movement sub-components are most susceptible to breakdown. Assessing this in relation to fatigue provides a method for establishing the utility of the Craig et al. (2000) model in determining the motor control abilities of the elite player and how these might change over the course of play.

The effects of fatigue or feelings of tiredness on golf putting performance is of particular interest given the physical demands of the sport and the known detrimental effects that fatigue has on a range of motor tasks. Wilkins et al. (2004) showed that exercise-induced fatigue is associated with greater errors in postural stability and that errors are most notable on complex balance tests. Lepers et al. (1997) also found significant decreases in postural stability after a 25 km run on complex, but not simple, balance tests, and other studies have shown that fatigue produces greater performance variability, slowed reaction time, and increased errors in a variety of sport performance measures (Lees, 2003). More specifically, Evans et al. (2008) demonstrated that fatigue produced by 40 min of golf putting practice induced significant changes in the full golf swing patterns that followed. Sensations of tiredness have also been found to adversely affect the accuracy of performance on fine perceptuo-motor tasks (Liebermann et al., 2002; Philip et al., 2003; Huysmans et al., 2008), co-ordination when throwing (Forestier and Nougier, 1998) and result in poor timing, general lethargy, and either a loss of concentration or a shift in the direction of attentional focus that would be detrimental to the task demands (Matthews, 1995; Davey et al., 2002). Whilst these findings indicate that fatigue could impact on the scaling of the golf putting stroke other research has shown that expert performers are able to compensate for the effects of fatigue by adjusting their movement patterns to maintain spatial accuracy (Aune et al., 2008). Therefore, there remains a need for further research into possible changes in how, or if, golfers change their putting actions for different putt lengths when they are fatigued, and to identify the levels of tiredness that might have to be generated before significant deterioration, or compensation, in their scaling performance is noted.

The current study had three aims. The first was to describe the ways in which a group of elite golfers systematically manipulated the amplitude, timing, and velocity profile of their downswing to achieve putts of different distances using the Craig et al. (2000) model as a framework. In particular we wanted to assess whether elite golfers used a simple strategy of manipulating the amplitude of the swing, as suggested by Craig et al. (2000), or whether they used more complex and individualized scaling strategies. The second aim was to assess the test–retest reliability of the Craig et al. (2000) model for a group of elite golfers. If this model is to be used in practice then it should be able to record behavior consistently but be sufficiently robust to accommodate minor fluctuations in performance. The relative stability of elite golfers’ putting actions provided a good opportunity to test the model. We predicted that if the model has sufficient sensitivity and retest reliability then the findings from testing the golfers at two different time points would be the same. The third aim was to explore the impact of fatigue on golf putting performance using the Craig et al. (2000) model. To do this we examined the effects of walking-induced fatigue on the scaling strategies used by the golfers.

## DEFINING SCALING STRATEGIES (TIME 1)

### METHOD

#### Participants

The participants were three female and three male elite amateur golfers (*M*_age_ = 19.33, *SD* = 1.50 years, and *M*_handicap_ = 1.0, *SD* = 0.63 strokes) who were part of a University International Sports Scholarship Program. Local ethical approval was granted for this study.

#### Materials

The study was carried out in a purpose built indoor artificial putting green (measuring 2 m by 5 m) that had four standard golf holes (diameter 10.8 cm) embedded at distances of 1 m, 2 m, 3 m, and 4 m from defined start positions at one end of the surface. The surface was covered in a green colored synthetic textile material with similar retarding characteristics to putting surfaces used in elite competition (USGA Stimpmeter reading of 3.05 m). A series of pilot tests showed that the proportion of energy loss at ball impact and the retarding force on the ball were relatively constant and linear regressions of putterhead velocity ($V_{c}^{2}$) against putt distance, where putts were not aimed at a hole (to mimic the conditions used by Craig et al., 2000), gave *r*^2^ values comparable to those reported by Craig et al. (2000).

The putt distances were chosen for this study matched those used by Craig et al. (2000), although the participants in the current study putted to golf holes that were embedded into the artificial putting surface as opposed to target zones. The movement of the putterhead was recorded using three Qualisys motion capture cameras sampling at 240 Hz with markers attached to the heel and toe of the putterhead. The cameras were set up to capture the three dimensional movements and used a suitable aperture and depth of field to generate a complete data stream for the putterhead for the backswing and downswing movements of the putting stroke. The downswing is responsible for applying force to the golf ball and begins when the velocity of the putterhead moves away from zero at the top of the backswing and comes to an end when the velocity of the putterhead returns to zero at the end of the movement. The calibration parameters were established by measuring the variation in the length of a calibration wand (600 mm) as it passed through the depth of field in which the putting stroke was performed. The difference between the maximum and minimum wand length recorded by the Qualisys system was 0.494 mm (0.08%) and the standard deviation in the wand length was.154 mm (0.025%). Each golfer used their own putter and supplied a golf ball that conformed to the cover type and compression of their choice during the period of data collection. During a set of calibration trials prior to the test participants attempted each of the putting distances from a pre-determined starting position so that the point of contact between the putterhead and the ball during impact could be identified and the distance between the putterhead and the golf ball at address could be ascertained.

#### Data processing methods

Data were analyzed using bespoke LabVIEW software. First data were filtered using a Gaussian filter with a sigma value of six. Then the start and end of the downswing movement of the putting stroke, and the point of impact between the putterhead and the golf ball (determined from the calibration trials) were noted. The start of the movement was marked as the first data point when the velocity was greater than zero and the end point was marked as the last data point before the velocity returned to zero. Using the downswing data for each putt several measurements were taken. These were: squared velocity of the putterhead at impact ($V_{c}^{2}$); proportion of time-to-impact from the start of the downswing (*P*_t_); the inverse of the downswing duration squared (1/*T*^2^); the amplitude of the downswing squared (*D*^2^); the shape of the velocity profile of the movement (*k*) as defined by Craig et al. (2000).

#### Procedure

Each golfer completed a period of familiarization and a set of calibration trials. They then performed 20 putts to each of the four holes that were located at distances of 1 m, 2 m, 3 m, and 4m. The ball was placed precisely on the starting position for each trial and the 80 putts were performed in a random order. The participants were allowed to rest between putting trials whenever they detected the onset of physical or mental fatigue. The participants typically performed a sequence of eight putting trials then sat down for a brief rest period before continuing with the data collection.

## RESULTS

Out of the 480 putts recorded 443 were successfully holed yielding a success rate of 92.3%. Only successful putts were included in the analyses of how the golfers controlled the putterhead velocity at ball impact. Unsuccessful trials were not included since the lack of success could be attributed to either an inappropriate force being applied to the ball or a directional error, or both.

To confirm that these elite golfers were skilled at controlling the velocity of the putterhead at ball impact descriptive statistics for their performances were examined (**Table 1**). The low standard deviations and coefficients of variation both within and between participants indicated that these elite golfers were consistent and skilled in their ability to control putterhead velocity at ball impact to achieve different putt distances.

**Table 1:** Mean, standard deviations (SD), and coefficients of variation (CV) for putterhead velocity ($V_{c}^{2}$ ms^-1^) at ball impact for each participant (P) and putt distance.

	1m			2m			3m			4m		
	*M*	*SD*	*CV*	*M*	*SD*	*CV*	*M*	*SD*	*CV*	*M*	*SD*	*CV*
P1	0.97	0.03	0.04	1.17	0.06	0.05	1.40	0.05	0.03	1.61	0.04	0.03
P2	1.01	0.04	0.04	1.18	0.05	0.04	1.34	0.06	0.04	1.59	0.04	0.02
P3	0.97	0.04	0.05	1.17	0.05	0.04	1.40	0.05	0.03	1.58	0.05	0.03
P4	0.90	0.06	0.06	1.08	0.07	0.06	1.30	0.04	0.03	1.53	0.05	0.03
P5	0.86	0.04	0.04	1.07	0.03	0.03	1.29	0.06	0.05	1.53	0.08	0.05
P6	0.98	0.04	0.04	1.13	0.04	0.03	1.33	0.04	0.03	1.58	0.06	0.04

**FIGURE 1:**
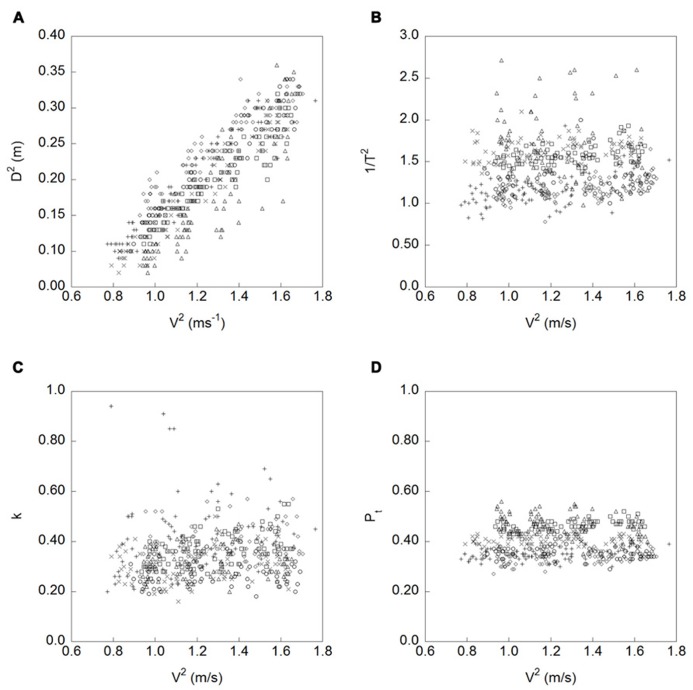
**Relationships between the velocity of the putterhead at impact ($V_{c}^{2}$ ms^-1^) and; **(A)** swing amplitude (*D*^**2**^), **(B)** swing duration (*1/T*^**2**^), **(C)***k* a constant that denotes the shape of the putterhead velocity profile (Lee, 1998), and **(D)** proportion of time from the start of the swing to impact (*P*_**t**_).** Each participant’s data is represented by a different symbol.

Analyses of the scaling variables proposed by Craig et al. (2000) were then undertaken to identify how each of the golfers controlled the putterhead velocity over the different putt distances. **Figure 1** displays the data and indicates that whilst the golfers seemed to be systematically varying the amplitude of the downswing to change the force of impact, the other three variables (*P*_t_, *1/T*^2^,and *k*) were not being held constant across the four putt distances. Multiple regression analyses were then carried out on the data for each golfer to determine if any of the four variables predicted the putterhead velocity at ball impact. Prior to this the data were checked to determine whether the data points within each series were independent, as this is an assumption of multiple regression analysis. To do this the autocorrelation within each data series was examined. This indicated that two of the data sets (P2 and P5) had significant first order autocorrelations. To remove the serial dependency within these data sets we used a whitening technique whereby by we created a lagged data series which we regressed onto the original data series and saved the unstandardized residuals. Additionally, we examined the collinearity amongst the predictor variables for each participant. Here we adopted a procedure of variables showing high bivariate correlations and variance inflation factor values greater than three were to be deleted from the model. This procedure was carried out for each golfer’s data but did not result in any variables being removed from the models.

The results of the multiple regression modeling are summarized in the top of **Table 2**. This showed that none of the golfers relied on a single variable strategy of movement scaling as hypothesized by Craig et al. (2000). Two different strategies emerged indicating some individual differences between these elite golfers. Two of the participants (P2 and P5) varied *D*^2^ and 1/*T*^2^ systematically to change the putterhead velocity over the four putting distances at baseline whilst the other four golfers (P1, P3, P4, and P6) used three variable models involving *D*^2^, 1/*T*^2^,and *P*_t_. The variable *k* did not appear in any of the models that were generated by the multiple regression analyses.

**Table 2:** Model summaries from the multiple regression analyses showing the standardized β for each of the predictors of putterhead velocity squared at ball impact for each participant’s successful putts.

			Standardized β			
	Model	Adjusted *r*^2^	*D*^2^	*1/T*^2^	*P*_t_	*k*
**Time 1**
P1	*F*(4,71) = 565.92, *p* < 0.001	0.970	0.91^**^	0.38^**^	-0.21^**^	0.03
P2	*F*(4,70) = 105.89, *p* < 0.001	0.858	0.89^**^	0.13^**^	0.03	-0.04
P3	*F*(4,65) = 376.44, *p* < 0.001	0.958	0.96^**^	0.18^**^	-0.14^**^	0.07
P4	*F*(4,71) = 331.24, *p* < 0.001	0.946	1.01^**^	0.16^**^	-0.10^**^	-0.02
P5	*F*(4,66) = 173.42, *p* < 0.001	0.913	0.96^**^	0.18^*^	0.01	0.01
P6	*F*(4,70) = 140.17, *p* < 0.001	0.889	1.07^**^	0.51^**^	-0.25^**^	0.03
**Time 2**
P1	*F*(4,65) = 479.09, *p* < 0.001	0.965	0.96^**^	0.27^**^	-0.17^**^	0.03
P2	*F*(4,62) = 372.83, *p* < 0.001	0.950	0.85^**^	0.25^**^	0.06	0.01
P3	*F*(4,71) = 341.68, *p* < 0.001	0.946	0.94^**^	0.26^**^	-0.18^**^	0.04
P4	*F*(4,51) = 321.48, *p* < 0.001	0.945	0.98^**^	0.18^**^	-0.03	-0.05
P5	*F*(4,70) = 252.00, *p* < 0.001	0.927	0.86^**^	0.20^**^	0.02	-0.01
P6	*F*(4,61) = 173.42, *p* < 0.001	0.897	1.06^**^	0.67^**^	-0.33^**^	0.02
**Time 3**
P1	*F*(4,60) = 146.38, *p* < 0.001	0.899	0.89^**^	0.25	-0.18	0.02
P2	*F*(4,64) = 685.81, *p* < 0.001	0.973	0.82^**^	0.48^**^	-0.02	0.05
P3	*F*(4,49) = 271.99, *p* < 0.001	0.933	0.95^**^	0.39^**^	-0.34^**^	0.02
P4	*F*(4,67) = 367.79, *p* < 0.001	0.949	0.96^**^	0.17^**^	-0.01	-0.07
P5	*F*(4,49) = 170.21, *p* < 0.001	0.900	0.86^**^	0.16^**^	0.08	-0.01
P6	*F*(4,64) = 119.92, *p* < 0.001	0.858	1.04^**^	0.56^**^	-0.16	-0.05

Time 1 data shows baseline scaling strategies, time 2 data was collected approximately 6 months later (pre-exercise), and time 3 data was also collected at 6 months when the golfers were fatigued post-exercise. *p < 0.05, **p < 0.01.

## DISCUSSION

These elite golfers were highly consistent in the velocity of the putterhead at impact that they generated for each of the four putt distances. However, there were substantive individual differences between the golfers and this was most noticeable with the 1 m putt distance where a range of “holing out” strategies can be used effectively in the real life setting (Pelz, 2000). Elite golfers can use a higher impact velocity with less allowance for the natural contours of the green, or use a slower impact velocity and allow the ball to follow the slope of the green into the hole. At the 1 m distance the participants mean putterhead velocity at impact ranged from.86 ms^-1^ to 1.01 ms^-1^ giving a 14.85% variation between the participants, whereas the difference between participants at 4 m was only 4.96% of the maximum mean velocity recorded. This was to be expected given that the efficacy of higher velocity strategies reduces as the probability of success diminishes (i.e., on longer putts; Landsberger, 1994; Penner, 2002). The style favored by each of the golfers on the shorter putts would most probably have emerged from the history of success and failure experienced throughout a playing career, even though the putts in this study were performed on an artificial surface with no lateral slope and in a non-competitive situation.

The multiple regression analyses showed that two different strategies were being used to scale the putting actions to putt to the different distances. One strategy was to systematically vary the swing amplitude and duration, whilst the other was to systematically vary amplitude, duration, and the proportion of the swing duration before the ball was struck. Swing amplitude (*D*^2^) was clearly the most dominate variable in performance strategies as it appeared in all of the models and generated the greatest standardized β scores. However, both of the scaling strategies identified here also incorporated the duration variable 1/*T*^2^, which is somewhat surprising given the amount of instructional texts that recommend a consistent downswing duration across all putt distances (Pelz, 2000). The results also revealed that four of the six golfers had systematic variations in the proportion of the swing duration before the ball was struck (*P*_t_) to achieve putts of different distances, although the standardized βs were small ranging from (*β* = -0.10 to *β* = -0.25). The individual differences in the way these elite players completed the putting tasks was not unexpected given the multitude of different putting strategies available to the skilled golfer and opportunity for self-expression and uniqueness that the putting stroke affords (Penner, 2002). Despite the fact that two of the participants seemed to favor a two-parameter method of scaling (P2 and P5) whilst the other four employed a three-parameter method of scaling (P1, P3, P4, and P6) the ability of each participant to generate a consistent putterhead velocity at impact for the four putt distances was similar (see **Table 1**). There were no distinctive differences between P2 and P5 and the other golfers in terms of their experience or skill level, and further research is needed to determine why golfers develop different scaling strategies and whether there is an optimum style for consistent elite performance.

## CONSISTENCY OF SCALING STRATEGIES (TIME 2) AND THE EFFECTS OF FATIGUE (TIME 3)

Having established the putting strategies used by these elite golfers at baseline, we then aimed to determine the extent to which the Craig et al. (2000) model could demonstrate test–retest reliability. The same group of elite golfers was re-tested on the identical putting tasks after a period of 6 months (time 2). A period of 6 months was chosen for this purpose as this is approximately the length of the competitive golf season and was thought to be sufficiently long for any carry-over effects from the first testing session to be minimized. Since none of the golfers had deliberately changed their putting action over this time it was predicted that the regression models would not significantly differ between time 1 and time 2.

Lastly, the study aimed to determine whether the fatigue associated with competitive play would influence the scaling strategy and motor control of these elite players. The same golfers were asked to repeat the putting test protocol before and after completing an individual prescription of treadmill walking that mirrored the levels of fatigue that golfers may experience during a round of competitive golf. Data for the repeat test prior to walking (time 2) and after walking (time 3) were collected in one testing session. If the model showed good test–retest reliability between times 1 and 2, then it was assumed that any changes in the scaling strategies found between times 2 and 3 would reflect either a fatigue-induced decline in motor control or a compensatory change in motor control patterns to accommodate the fatigued state. If the changes were a mark of a decline in motor control then those participants who demonstrated these would be expected to report large increases in fatigue levels and show drops in their success rates. On the other hand, if changes in the scaling strategies were compensatory behaviors it would be expected that success rates would be maintained even as fatigue levels increased.

### METHOD

#### Participants

The same six elite amateur golfers who participated in the first part of the study agreed to participate in this phase.

#### Materials and data processing

The same materials and data processing methods were used to capture the putting performance data. The level of pre-putt general fatigue was measured before each trial using a self-report visual analog scale as this has been suggested as the most appropriate method of measurement in experimental settings (Matthews, 1995; Matthews and Desmond, 2002). The scale consisted of a 10 cm horizontal line with the verbal descriptors of fatigue state being “extremely low,” “moderate,” and “extremely high.”

#### Procedure

First each participant performed individually and completed 80 putting trials (20 to each of the four distances) as in the data collection at time 1. Before each attempt a pre-putt measure of fatigue was taken using the visual analog scale. This was completed approximately 10 s prior to each putt. Participants were told they could rest at any time, and they should take a break if they detected the onset of fatigue. The participants then completed 60 min of treadmill walking (at 6.5 km/h) at an incline that generated a heart rate response of 70% of maximum heart rate. This workload required the participants to walk a distance of approximately 6.5 km which is consistent with the distance covered by a golfer during a round of competitive play (Broman et al., 2004). Following this period of exercise, the participants performed a single block of 100 consecutive golf putts to the same four golf holes as before in a random order. During this test the participants were not permitted to rest and they reported their level of pre-putt fatigue approximately 10 s before each of the putting trials.

## RESULTS

A paired *t*-test was carried out to examine the number of putts holed between time 1 and time 2 and showed no significant difference existed between the respective success rates (time 1; *M* = 92.29%, *SD* = 3.00, time 2; *M* = 91.66%, *SD = 2.81*, *t*(5) = 0.31, *p* = 0.77). A series of *t*-tests were also undertaken on the data from each golfer to compare the putterhead velocity at ball impact for the two time points and these revealed no significant differences in performance over the 6 month period for any of the putting distances. This was the case both with and without Bonferroni corrections.

The same procedures for assessing autocorrelation and collinearity were applied prior to the multiple regression analyses to determine predictors of putterhead velocity at ball impact for each player. Data are summarized in the top and middle sections of **Table 2**, and comparing the regression models from baseline (time 1) to 6 months later (time 2) it seemed that five of the six golfers had the same pattern of behavior when testing was repeated, and only P4 appeared to show a change in their scaling model where the proportion of the downswing duration from the start of the movement to impact (*P*_t_) was a significant predictor of putterhead velocity at impact at baseline but it was not a significant predictor 6 months later. To test if the unstandardized betas and standard errors in the regression models were statistically different between times 1 and 2, the *t*-test proposed by Edwards (1984) was used. This showed no significant differences for any of the predictor variables for any participant, including the proportion of the downswing duration from the start of the movement to impact (*P*_t_) at times 1 and 2 for P4 (*t* = 1.29, *p* > 0.05).

Since these results demonstrate that the Craig et al. (2000) model was found to have good test–retest reliability for these golfers, the effect of the walking intervention (fatigue) was then assessed. A paired samples *t*-test was carried out to determine if there were changes in the self-reported fatigue scores before and after the prescription of exercise. The results showed a significant increase in fatigue from pre to post-exercise conditions (pre-exercise; *M* = 16.80, *SD = 17.35*, post-exercise; *M* = 47.60, *SD = 11.23*, *t*(5) = 6.88, *p* < 0.001). Individual *t*-tests were also carried out on each golfer and demonstrated that all the golfers reported significant increases in fatigue after exercising (*p* < 0.01). There was also a significant decrease in the percentage of putts that were holed between pre and post-exercise (*t*(5) = 4.08, *p* < 0.01), with all the golfers holing less putts and an overall drop in the success rate from 91.66% (*SD* = 2.81%) to 82.30% (*SD* = 4.90%) when fatigued. However, there was no significant correlation between changes in success and changes in fatigue (*r* = 0.12 *p* = 0.82).

The same procedures for regression modeling were used for the post-exercise data, and the results were compared to the pre-exercise data (summarized in the middle and bottom sections of **Table 2**). Edwards’s (1984) test of differences between unstandardized betas showed that three of the golfers maintained the same performance scaling strategy, but the other three showed changes post-exercise. Both P1 and P6 used a simplified scaling model when they were fatigued. From **Table 2** it appears that P1 changed from a scaling strategy where *D*^2^, 1/*T*^2^, *P*_t_ predicted putterhead velocity when not fatigued to a strategy of varying just the swing amplitude (*D*^2^) when fatigued. The Edwards’ test showed a significant reduction in post-exercise unstandardized betas for swing amplitude for P1 (*t* = 2.18, *p* < 0.05). The regression model for P6 showed that *D*^2^, 1/*T*^2^,and *P*_t_ predicted putterhead velocity when not fatigued, but only *D*^2^ and 1/*T*^2^ were significant predictors of putterhead velocity when they were fatigued. The pre to post-exercise reduction in the unstandardized betas for *P*_t_ was significant for P6 (*t* = 1.97, *p* < 0.05). P3 also showed a significant change in their scaling strategy whereby the extent to which duration (1/*T*^2^) predicted putterhead velocity significantly increased from before to after exercise (*t* = 2.51, *p* < 0.05).

Finally, we examined the putterhead velocity at ball impact across the three time points. As shown in **Table 3** between the first testing session (time 1) and the repeat session 6 months later (time 2) there was little variation in performance. However, after exercise (time 3) there was an increase in putterhead velocity for the 1 m, 2 m, and 3 m putts. We conducted a two way (time × 3, putt distance × 4) ANOVA on this data which showed the main effect of time to be approaching significance [*F*(2,10) = 3.94, *p* = 0.055, *r* = 0.53] but the interaction effect was non-significant [*F*(6,30) = 1.14, *p* = 0.36, *r* = 0.19].

**Table 3:** Means and standard deviations for the velocity of putterhead (ms^**-1**^) at the point of impact for each of the four putt distances at time 1 (baseline), time 2 (6 months after baseline and prior to exercising), and time 3 (post-exercise).

	1 m putts		2 m putts		3 m putts		4 m putts	
	*M*	*SD*	*M*	*SD*	*M*	*SD*	*M*	*SD*
Time 1	0.95	0.06	1.13	0.05	1.34	0.05	1.57	0.03
Time 2	0.95	0.06	1.14	0.05	1.36	0.04	1.58	0.03
Time 3	0.97	0.06	1.16	0.05	1.37	0.05	1.57	0.03

Individual success rates were then examined to assess whether the changes shown by P1, P3, and P6 were related to motor control decline or compensation for the self-reported fatigue levels (**Table 4**). P1 showed the lowest success rates at time 2 (86.25%) and time 3 (80.00%) and the highest increase in fatigue (46.84 points), which suggested that their change in scaling strategy could have been due to a decline in motor control. Similarly, P3 showed the largest decrease in success rate (20%) and the second largest increase in fatigue (41.14 points) which also suggested that their changes could have been due to a decline in motor control. However, P6 showed a relatively smaller drop in success (8.75%) but also had a relatively small increase in fatigue (17.16 points) making it difficult to attribute the change in their scaling strategy to either a decline in motor control or a compensation to cope with the effects of fatigue.

**Table 4:** Success rates for all three testing sessions and fatigue scores before (time 2) and after walking (time 3).

	Success rate (%)			Fatigue score	
	Time 1	Time 2	Time 3	Time 2	Time 3
P1	93.75	86.25	80.00	1.33	48.20
P2	93.75	92.50	85.00	8.89	41.09
P3	86.25	93.75	73.75	15.30	56.44
P4	93.75	91.25	87.50	7.58	40.34
P5	93.75	92.50	82.50	50.20	64.66
P6	92.50	93.75	85.00	17.53	34.69

## DISCUSSION

From the data collected at time 1 it was evident that elite golfers do not have a uniform strategy for scaling their putting actions to different distances. Changing the amplitude of the swing was a central feature of their strategies, but these golfers were also systematically varying the overall duration of the swing and the proportion of time from the start of the swing to impacting with the ball. Looking across the first two data collection time points, the golfers were able to maintain their success rates (number of putts holed) between baseline (time 1) and the 6 month follow-up (time 2), and there were no significant differences between the unstandardized betas of the predictor variables for any of the golfers. However, it should be noted that whilst not statistically significant the model for P4 did show a trend toward change. These findings indicate that the Craig et al. (2000) model can reliably track behavior over a period of time, and suggests that it is not overly sensitive to minor fluctuations in performance. This implies that any changes noted following exercise were unlikely to be the result of natural variations in behavior.

Comparing the pre and post-exercise (time 2 and time 3) data showed that the number of successful putts decreased for all of the players when they were fatigued, and three of the six participants showed noticeable changes in how they controlled the putterhead velocity. Both participants (P1 and P6) simplified their performance scaling strategy when they were fatigued, and P3 showed a significant increase in *1/T*^2^ as a predictor after exercise. Trends were also noted in the putterhead velocity at impact which indicated an increase in velocity for the shorter distances when the golfers were fatigued. However, it should also be noted that there were differences in the fatigue scores which makes interpreting the data for some golfers more difficult. Looking at the differences in fatigue scores suggests that P1 (46.8 point change) and P3 (41.14 point change) were the most influenced by the walking intervention, and P6 with a 17.17 point change was less influenced, even though this was a significant increase. Interestingly, P5 reported a very high baseline fatigue level and the highest post-exercise level at 64.66 points. Since we did not record fatigue levels at baseline (time 1) it is not possible to determine if this was usual for this golfer, however, the stability in their success rates between times 1 and 2 (1.25% change) and the drop in success between times 2 and 3 (10% decline) suggests that the walking intervention did impact on their performance even though this was not evident in their scaling strategy. The remaining two participants P2 and P4 showed comparable changes in fatigue levels (32.20 and 32.77 point changes, respectively) and relatively small drops in success rates (7.5% and 3.75%) indicating that whilst there change in fatigue levels were significant they may not have been extreme enough to induce changes in scaling strategies.

The decrease in success rate when fatigued is consistent with the wealth of evidence that links increased physical fatigue to changes in performance on a range of motor tasks (Liebermann et al., 2002; Philip et al., 2003; Evans et al., 2008; Huysmans et al., 2008), but whether the methods used to scale the putting action by P1, P3, and P6 reflects a compensation strategy in line with Aune et al. (2008), or the start of decline in performance similar to that discussed by Lees (2003) could not be categorically established for all three golfers. Interestingly, even though the predictors in the models used to achieve different putterhead velocities changed when these golfers were fatigued, the degree to which the models accounted for the variance in the data (as shown by the adjusted *r*^2^ values) remained similar. Moreover, these fatigue related changes were evident in their successful putts indicating that even the outcome goal was achieved their movement patterns were not consistent. Whilst the data for P1 and P3 pointed to a decline in motor control, for P6 the findings may be indicative of a compensation strategy being used, as alluded to by Aune et al. (2008). For example, it may be that there were significant differences in the timing of P6’s swing at the initial stage of the movement, which diminished as the act unfolded, and had completely receded at the moment of impact. These are tentative conclusions though and further testing would be required to confirm this.

The findings of this study demonstrate subtle changes to complex patterns of motor control that would not have been apparent from the analysis of the basic outcome performance measures used in some of the previous studies (DeJong et al., 1998; Lorist et al., 2002; Matthews and Desmond, 2002). The performance measures used here provide an insight into the way in which fatigue may impact on the golf putting process and may provide some suggestions for elite golfers who wish to maintain their level of performance during conditions of fatigue. The variations in performance that were noted in this study were evident when the putts were holed successfully, however, no analysis of the unsuccessful putts was undertaken, due to the small number of trials on which failures occurred. Therefore, it is possible that poor putterhead velocity control contributed to these failures too, although other sub-components such as reading the line or aligning the putter face to the target line may have been more significant factors. Whilst further research into the cause of these errors is needed, the artificial surface used in the current study removed the requirement for the golfers to “read the line of the putt” and remained stable between the non-fatigued and fatigued conditions, which suggests that the most likely sources of error in the unsuccessful trials were in the force applied to the ball or the alignment of the putterface as the golfers prepared to putt. The direction of the golf ball immediately post-impact is known to be determined by the position of the putterhead as the club makes contact with the ball. Thus, it is possible that during fatigue, the players prioritized their available attentional resources to monitoring the amplitude and temporal control of the action, and this may have reduced the resource required for the correct alignment of the putterface as a consequence. Such attentional changes are well documented throughout the sport psychology literature and directing attention more internally (i.e., on the mechanics of the stroke) during conditions of fatigue has often been suggested as an explanation for performance failure (Wulf, 2013). The implication here would be that when a golfer detects fatigue when putting they should be aware of the need to prioritize alignment accuracy as well as the amplitude and temporal control of the stroke for best results. This would ensure that emphasis is given to the intended movement effect as well as the production of the correct force of impact.

The findings of this study have implications for Craig et al.’s (2000) predictions about how action scaling is achieved, and more generally for their theoretical framework for how actions are controlled. The broader theoretical stance that Craig et al. (2000) adopt is, in part, similar to the concept of an internal model, since they propose that golfers use an internally generated spatio-temporal template which is used to initiate and guide the swing action. The nature of this internal guide, for the purposes of golf putting, is adapted by altering some or all of the four parameters in the Craig et al. (2000) model as described above. However, where Craig et al. (2000) take a different theoretical stance to most motor control theorists is in the role that they believe perception plays in guiding actions. They adopt Lee’s (1998) approach of direct perception which proposes that all information gathered thorough the senses has a specific spatio-temporal form (known as tau), and that actions are controlled by regulating the closure of the gap between an effector and its goal by using tau information which is perceived directly from the environment. Thus, the belief is that the perceptual system, internal model, and motor system are linked by a common form of information.

The notion of direct perception-action coupling has been heavily criticized, as demonstrations that perceptual information, other than tau, can be used to guide actions (for example, Land’s commentary on Tresilian, 1997, 1999; Brouwer et al., 2003; Lee et al., 2009). The current study adds to this debate by demonstrating that the formation of an internal guide for a putting action can involve the complex manipulation of information about the amplitude of the movement, the proportion of time from the start of the swing to ball impact and the reciprocal of the movement duration squared. Taking P1’s regression model at time 1 as an example, it can be seen that three variables predicted the putterhead velocity at ball impact. This implies that when putting P1 had formed an internal guide by choosing appropriate values for each of these three inter-linked variables. This seems to add a layer of complexity that is not clearly articulated by a direct perception-action approach. Moreover, it could be argued that putting is a relatively simple skill and other more complex skills would engender even greater levels of complexity. Whilst this does not detract from the use of Craig et al.’s (2000) model to describe putting behavior it does suggest that the general tau theory is not yet sufficiently complex to understand how internal guides are produced that allow for putting actions to be controlled.

It should also be noted that this study has limitations and the findings suggest recommendations for future work. The small sample size limits the statistical power of the group based analysis that was carried out on the putterhead velocity at impact. However, the regression analyses point to considerable inter-individual variability in scaling strategies which may eventually be more useful for the truly elite golfer. This variability suggests that group based designs may not be the most effective way to study putting behavior in either elite or non-elite athletes as a variety of different styles are likely to exist. At baseline two different patterns of behavior emerged from these six golfers, and it is quite likely that more patterns will emerge with further study. Further work will also allow us to determine if there are advantages to using particular strategies in terms of consistency of performance and coping with the pressures of competitive play.

A second limitation of this study was the artificial nature of the putting green that was used. This was chosen to standardize the putt distances and for ease of recording the movements, however, it leaves the question as to how generalizable the findings are for golfers playing in match conditions. Further consideration also needs to be given to the degree of sensitivity to change that the Craig et al. (2000) model possesses. We have argued that the stability shown in the regression models between times 1 and 2, and the changes seen in some golfers before and after exercise, provide preliminary evidence that the model is both reliable and sensitive to meaningful changes, rather than small fluctuations in behavior. However, this is based on only three measurements per participant, and the addition of more repeated testing would provide a more accurate indication of reliability and sensitivity to change.

In summary, the Craig et al. (2000) model provided a useful and informative assessment of the scaling methods used by six elite golfers which were varied and more complex than originally predicted. The study showed that fatigue was associated with a decrease in the task outcome (the number of putts that were holed), and subtle changes in the motor control of the skilled putting act even when they holed the putt. For half of the players, fatigue did not change the strategy they used to produce putts of different distances, but there was some indication that overall they were hitting the ball harder when fatigued. Used in conjunction with other measures, assessing the scaling strategy used across different putts could provide practitioners with a useful means of monitoring and assessing golf putting behaviors in a variety of situations that relate to competitive play. The advantages of using this model is that it provides a means of comparing patterns of motor control between both similar and different putt lengths of individual golfers. It highlights the individual differences amongst elite golfers and provides a detailed analysis of their motor control which will allow for tailored interventions to be devised. The disadvantage of using this model, as with many other detailed measurements of movement control, is that it is ideally suited to an indoor putting environment and will be more difficult to apply in the field.

## AUTHOR CONTRIBUTIONS

John F. Mathers and Madeleine A. Grealy designed the studies. John Mathers recruited the participants, collected the data, and analyzed the results. John Mathers and Madeleine Grealy contributed to the writing of the manuscript.

## Conflict of Interest Statement

The authors declare that the research was conducted in the absence of any commercial or financial relationships that could be construed as a potential conflict of interest.
