# Corrigendum – “Motor control strategies and the effects of fatigue on golf putting performance”

**DOI:** 10.3389/fpsyg.2014.01583

**Published:** 2015-01-20

**Authors:** John F. Mathers, Madeleine A. Grealy

**Affiliations:** ^1^School of Sport, University of StirlingStirling, UK; ^2^School of Psychological Sciences and Health, Humanities and Social Sciences, University of StrathclydeGlasgow, UK

**Keywords:** corrigendum, motor, control, fatigue, golf, putting

The following text replaces the fifth paragraph of the Introduction section of the original article:

Whilst manipulating the amplitude of the swing provides a simple method of varying putt distance, other systematic variations in either Pt,1/T2,or k could produce similar outcomes. These four variables could be manipulated individually or in combination with each other, to achieve different putt distances. Assessing this in relation to fatigue provides a method for establishing the utility of the Craig et al. (2000) model in determining the motor control abilities of the elite player and how these might change over the course of play.

For example, Fairweather et al. (2002) found that elite golfers scaled the putterhead velocity at ball impact for longer putt distances by increasing the amplitude of the swing and decreasing the duration of the swing. This interactive manipulation would translate into relatively small alterations to the movement variables and could preserve the pro-portion of the swing duration before the ball is hit (Pt) and the shape of the velocity profile of the movement (k), resulting in them remaining relatively constant. This would be consistent with previous work that has emphasized the importance of preserving the temporal relationships of intra-movement segments within movement scaling (Whiting, 1985), and by those who propose that overall movement duration is an invariant characteristic within a skilled movement class (Schmidt, 1975, 2003). To date though there is limited research on whether elite golfers alter their putting actions using a uniform method, or whether certain methods of scaling make the player more or less susceptible to making errors when they experience feelings of anxiety or fatigue. This is somewhat surprising given the on-going quest for valid, reliable, and sport specific performance assessments against which coaching and practice interventions can be evaluated (Bishop, 2008; Drust, 2010). Moreover, if competitive conditions are associated with changes in motor control in the elite player, it would be important to identify which of the movement sub-components are most susceptible to breakdown.

## Conflict of interest statement

The authors declare that the research was conducted in the absence of any commercial or financial relationships that could be construed as a potential conflict of interest.
